# Correlates of substance use in a large naturalistic cohort of young people with early and emerging psychosis

**DOI:** 10.1007/s00127-023-02436-w

**Published:** 2023-02-20

**Authors:** D. El-Hage, C. X. Gao, G. Bedi, A. Guerin, S. Francey, H. Stavely, D. Rickwood, N. Telford, P. McGorry, A. Thompson, Ellie Brown

**Affiliations:** 1 Centre for Youth Mental Health, University of Melbourne, Parkville, VIC Australia; 2 Orygen, 35 Poplar Road, Parkville, VIC Australia; 3 School of Public Health and Preventive Medicine, Monash University, Clayton, VIC Australia; 4 Headspace National, Melbourne City, VIC Australia; 5 University of Canberra, Bruce, ACT Australia

**Keywords:** First-episode psychosis, Ultrahigh risk, Youth, Substance use, Tobacco, Early intervention

## Abstract

**Background:**

Substance use remains a barrier to recovery for young people accessing early intervention services for psychosis. While correlates of use have been explored in populations experiencing a first episode of psychosis (FEP), sample sizes have been small and less research assesses cohorts at ultrahigh risk of psychosis (UHR).

**Methods:**

This study uses data from a naturalistic cohort including UHR and FEP participants (*N* = 1252) to elucidate clinical correlates of use in the past 3 months of any illicit substance, amphetamine-type stimulants (ATS), cannabis, and tobacco. Moreover, network analysis based on use of these substances and additionally alcohol, cocaine, hallucinogens, sedatives, inhalants, and opioids was completed.

**Results:**

Young people with FEP used substances at significantly higher rates than those at UHR. High concurrence of use was seen between substances. In the FEP group, participants who had used any illicit substance, ATS, and/or tobacco had increased positive symptoms and decreased negative symptoms. Young people with FEP who used cannabis had increased positive symptoms. In the UHR group, participants who had used any illicit substance, ATS, and/or cannabis in the past 3 months showed decreased negative symptoms compared to those who had not.

**Conclusion:**

A distinct clinical picture of more florid positive symptoms and alleviated negative symptoms seen in those who use substances in the FEP group appears muted in the UHR cohort. Treating young people at UHR in early intervention services represents the earliest opportunity to address substance use early to improve outcomes.

**Supplementary Information:**

The online version contains supplementary material available at 10.1007/s00127-023-02436-w.

## Introduction

Early intervention services for young people experiencing a first episode of psychosis (FEP) are now well established as an effective and cost-efficient treatment strategy [9, 37]. Many of these services now recognise the benefit of treating individuals at ultrahigh risk of psychosis (UHR), defined by having one or more of: attenuated psychotic symptoms, brief limited intermittent psychotic symptoms, or trait vulnerability coupled with recent functional decline [35, 39]. Transition to FEP occurs in 25% of young people identified as being at UHR status after 3 years, rising to 35% by 10 years from initial assessment [16]. The UHR designation is also known as clinical high-risk (CHR), or the at-risk mental state for psychosis (ARMS) [22].

Longitudinal studies of young people with FEP or at UHR who access early intervention services have routinely shown improvements in clinical symptomatology alongside personal and occupational functioning [1, 9, 15]. However, relapse remains common in those with FEP, occurring in around 30% by 1 year and up to 83% at 7.5 years after treatment entry [3, 10]. One factor associated with high relapse rates in FEP populations is substance use and substance use disorder [3]. Substance use typically occurs at rates at least double those of the wider population in both FEP [7, 13, 14] and UHR cohorts [2]. Among those with FEP, substance use is associated with a higher positive symptom burden, lower levels of personal and occupational functioning, and treatment resistance [8, 28, 32, 34]. Early cessation of substance use has been found to be correlated with reversal of these outcome deficits [38].

There is uncertainty surrounding psychiatric symptom levels and substance use in UHR populations. On one hand, a recent systematic analysis of five studies found only one study demonstrated more unusual thought, attenuated psychotic symptoms and negative symptoms to be associated with substance use [19]. On the other hand, higher levels of positive psychotic symptoms have been described amongst those who use cannabis in UHR cohorts in a meta-analysis of 30 studies [12]. Overall associations between substance use, symptomatology, and functioning in UHR populations remain to be satisfactorily elucidated.

Encouragingly, many young people involved with early intervention services reduce and abstain from substance use even when it is not a specific focus of the services [5, 9]. Compared to those who continue to use, those who do reduce their use demonstrate better medication compliance and treatment engagement alongside lower rates of relapse and fewer negative symptoms at 10-year follow-up [25, 38]. In fact among an FEP cohort those who stopped using substances early in the course of their illness had better long-term outcomes than those who had never used [38].

While half of young people using substances at the time of entry into early intervention services will reduce use after 12 months of engagement, half will not [5]. There is a dearth of guidelines for identifying individuals at risk of persisting use or optimal approaches for specific interventions to reduce substance use in the context of early intervention services. Treatment targeting use reduction is easily overlooked in a system with limited resources and competing priorities. A more holistic understanding of patterns, factors associated with, and symptomatic correlates of substance use, the most salient drugs to target, and distinctions among treatment cohorts would aid in the formulation of effective treatment protocols for those who need more targeted treatment.

Using clinician-collected data of consenting participants diagnosed with either UHR or FEP at the time of initial presentation to early intervention services across Australia, this analysis investigates:

- Past 3-month substance use prevalence for UHR and FEP cohorts.
- Correlations between substance use and symptomatology, functioning, and psychological distress.
- Distinctions between the UHR and FEP cohorts in substance use prevalence, patterns, and symptomology correlates.
- Internal associations between the use of studied substances within these samples, measured using network analysis.

## Methods

### Setting and study design

Data were collected from *headspace* Early Psychosis (hEP) services delivered at 14 treatment centres across Australia. Treatment for young people diagnosed with UHR or FEP at these sites is delivered based around the early psychosis prevention and intervention centre (EPPIC) model, which comprises 16 core components of care [33]. This is a baseline cohort not yet exposed to treatment, and so many participants are not formally diagnosed nor prescribed medication. Duration of care in these hEP services is recommended as a minimum of 2 years for FEP patients and 6 months for UHR patients.

Data used in this analysis is from the minimum data set collected by hEP clinicians as part of routine baseline data collection at the time of presentation. The study design is outlined in more detail elsewhere [11]. In brief, inclusion criteria of this study include age between 12 and 25 years and admission to one of the hEP services between 19th of June 2017 and the 30th of September 2019 with either a FEP or identified as being at UHR status, defined according to the Comprehensive Assessment of At Risk Mental States (CAARMS) criteria [40]. Data from one service cluster were excluded due to collection issues. Data were collected for each distinct treatment period that participants spent within the service, also known as ‘episodes of care’; however only data from the first presentation were used in these analyses. Participants’ age, self-reported gender, cultural and linguistic diversity status, Aboriginal and Torres Strait Islander status, and engagement with education/employment/training were collected at intake into headspace services. In the current analysis, these data were included as potential confounders only. They have been presented previously [11].

### Clinical outcomes

Substance use was assessed using the WHO ASSIST [24]. Non-medical use of alcohol, tobacco, cannabis, amphetamine-type stimulants (ATS), cocaine, hallucinogens, opioids, inhalants, and other substances was self-reported by participants. In the ASSIST, ATS includes any amphetamine derivative stimulant including methamphetamine, amphetamine (and its isomers), and 3,4-methylenedioxymethamphetamine (MDMA). Hallucinogens include lysergic acid diethylamide (LSD), psilocybin and mushrooms containing it, and synthetic hallucinogens such as NBOMes. The questionnaire asks individuals about how often they have used substances in the previous 3 months with potential responses being ‘never’, ‘once or twice’, ‘monthly’, ‘weekly’, or ‘daily or almost daily’. For regression and network analyses, responses were dichotomised to indicate either presence or absence of substance use in the previous 3 months.

Psychiatric symptoms were rated by clinicians according to the extended Brief Psychiatric Rating Scale (BPRS-E) and separated into positive and negative symptom subscales [18]. This scale rates a treating clinician’s perception of the severity of 24 distinct psychiatric symptoms with the total score range from 24 to 168. Two subscales have been used to assess positive and negative symptoms of psychosis. The BPRS-psychosis subscale (with a score range from 4 to 28) comprises four items: suspiciousness, hallucinations, unusual thought content, and conceptual disorganisation to measure the positive symptoms of psychosis. The BPRS-negative subscale (range from 3 to 21) comprises three items: blunted affect, emotional withdrawal, and motor retardation and is used to assess the negative symptoms of psychosis.

The Kessler Psychological Distress Scale (K10, 10 item ranging from 10 to 50) is a self-report measure used to assess subjective psychological distress [4].

Personal and occupational functioning was assessed with the Social and Occupational Functioning Assessment Scale (SOFAS) [21] which is rated from 0 to 100. For example, no interpersonal conflict and good occupational function would be rated at 100 with impairment in functioning denoted by a reduction in score. A score of 50 indicates “serious impairment across social, occupational, or school functioning (e.g. no friends, unable to keep a job)”.

### Ethics

All young people included in this study provided informed consent for their data to be used for service evaluation. Ethics approval was granted by the University of Melbourne human research ethics committee (ref: 2021-20371-13617-3).

#### Statistical analysis

Analyses were conducted using **R** version 4.0.2 (2020-06-22). Descriptive statistics were first used to describe characteristics of the young people included in this study. A range of analyses were subsequently conducted to understand detailed drug use profiles and associated correlates, as described below.

To facilitate readability of results tables, one decimal place is used for results under 10% and for *p* values over 0.1. Two decimal places are used for *p* values between 0.1 and 0.05, and three decimal places are used for *p* values less than 0.05.

#### Prevalence of drug use

Prevalence of the use any illicit substance, cannabis, ATS, and tobacco in the past 3 months were examined for FEP and UHR and stratified by age and gender subgroups. Comparison between UHR and FEP frequency of use was completed with Chi-square test.

#### Logistic regression

To evaluate associations between substance use patterns and clinical and functional measures, univariate and multivariate logistic regression models were performed. The use of any illicit substance, ATS, cannabis, and/or tobacco in the last 3 months were used as outcome variables and clinical outcomes (BPRS-psychosis, BPRS-negative, K10, and SOFAS) were predictors.

This study focussed on psychotomimetic substances cannabis and ATS and on exploring the link between tobacco and psychosis. Unlike these substances, alcohol has not been found to correlate with age of first psychotic symptom [31] and requires specific questions needed to establish binge drinking behaviour to differentiate from light recreational users, not a focus of this analysis. Similarly, the non-medical use of sedatives, which 16% of the UHR group had used, was not specifically investigated. Sample sizes for people who use inhalants, opioids, and hallucinogens were small (2.9%, 5.8%, and 11% of the UHR group with use in the past 3 months respectively) and so these substances were also not included for in depth analyses.

Demographic variables including age, gender, sexual orientation, education and employment were controlled for as potential confounders. Multiple imputation using chained equation (MICE) was used to address missing data, as per data management protocols [10].

#### Network analysis

Network analysis was used to elucidate interrelations between substances and to show qualitative differences in patterns of substance use between the UHR and FEP cohorts. To facilitate this analysis, substance use frequencies were dichotomised (“Yes/ “No” in the past 3 months). The network analysis first involves the estimation of pairwise associations between all drug use using tetrachoric correlations (${r}_{\mathrm{t}}$). A multidimensional scaling (MDS) network plot was used subsequently to visualise the correlations (${r}_{\mathrm{t}}$) on a two-dimensional space. These plots have a direct graphical interpretation, with shorter distance between nodes representing a stronger association. This graphical representation thus provides an overview of possible clusters and overall connectivity between the variables allowing for visual appreciation of associations between substances. This approach facilitates the interpretation of qualitative patterns of substance use between groups, and allows the inter-association of one substance with many others to be appreciated visually. Venn diagrams were also used to demonstrate overlaps between tobacco, cannabis, and ATS use in the UHR and FEP group separately.

## Results

### Demographics of study samples

In total, data from 609 UHR and 643 FEP participants were included in these analyses. The mean age of young people with UHR status was 17.5 (SD 3.0) and FEP was 19.7 (SD 2.7) years. Cohort characteristics can be seen in Table S1 in Supplementary Information and have been described elsewhere [11]. At baseline, the two groups had similar scores on the SOFAS, but those with UHR status scored significantly higher on psychological distress, with a mean K10 of 31.3 (SD 8.7), vs a mean K10 of 24.5 (SD 8.9) in the FEP group. Positive symptoms were slightly elevated in the FEP cohort with a mean BPRS-E psychosis scale score of 9.9 (SD 5.0), while the mean score was 8.5 (SD 3.4) in the UHR group. Negative symptoms were similar between cohorts (supplementary table S1).

### Prevalence of substance use

The proportion of patients using each substance category was first compared between cohorts (see Table 1 for UHR and FEP prevalence comparisons). The most common substances used by both cohorts were cannabis and tobacco. ATS, cannabis, tobacco, and any illicit substance were all more commonly used in the FEP than the UHR cohort (*p* < 0.001) with demographic variables such as age and gender controlled for.

**Table 1:** Past 3 months’ substance use comparison between UHR and FEP cohorts

Frequency	UHR, *N* = 622	FEP, *N* = 643	UHR, *N* = 622	FEP, *N* = 643
	*n* (%)	*n* (%)	*n* (%)	*n* (%)
	Tobacco; *p*-value^#^ < 0.001		Cannabis; *p*-value^#^ < 0.001	
Never	340 (58%)	243 (40%)	330 (56%)	254 (42%)
Once or twice	56 (9.5%)	64 (11%)	73 (12%)	66 (11%)
Monthly	16 (2.7%)	15 (2.5%)	28 (4.8%)	43 (7.1%)
Weekly	27 (4.6%)	28 (4.6%)	64 (11%)	81 (13%)
Daily or almost daily	149 (25%)	256 (42%)	93 (16%)	161 (27%)
	ATS; *p*-value^#^ < 0.001		Any illicit^*^; *p*-value# < 0.001	
Never	494 (84%)	443 (73%)	316 (54%)	234 (39%)
Once or twice	55 (9.4%)	80 (13%)	76 (13%)	69 (11%)
Monthly	25 (4.3%)	31 (5.1%)	31 (5.3%)	45 (7.4%)
Weekly	10 (1.7%)	32 (5.3%)	66 (11%)	84 (14%)
Daily or almost daily	4 (0.7%)	17 (2.8%)	99 (17%)	174 (29%)

^*#*^Pearson's Chi-squared test. Missing data for UHR include 21 for tobacco, cannabis, amphetamine-type stimulants (ATS), and any illicit substance Missing data for FEP include 38 for tobacco, 39 for cannabis, 41 for ATS, and 37 for any illicit substance

*Highest frequency of any reported illicit substance

### Multivariate analysis of the relationships between substance-specific effects, symptoms, and functioning

In the FEP cohort, the use of any illicit substance predicted higher levels of positive symptoms (OR = 1.06, 95% CI 1.02–1.11) and lower levels of negative symptoms (OR = 0.92, 95% CI 0.86–0.99) compared to non-use. The same pattern in symptomatology was seen with ATS use (positive symptoms OR = 1.08, 95% CI 1.02–1.12; negative symptoms OR = 0.92, 95% CI 0.86–1.00) and tobacco use (positive symptoms OR = 1.05, 95% CI 1.00–1.09; negative symptoms OR = 0.92, 95% CI 0.87–0.98). For cannabis use, higher levels in positive symptoms was seen in isolation (OR = 1.05, 95% CI 1.01–1.10) without any difference observable in negative symptoms.

In the UHR group, lower levels of negative symptoms were associated with the use of any illicit substance (OR = 0.90, 95% CI 0.82–0.98), with ATS (OR = 0.87, 95% CI 0.76–0.99), and with cannabis (OR = 0.91, 95% CI 0.83–0.99). No significant associations with positive symptom levels were found with the use of any substance in the UHR group. Neither symptoms of psychological distress (K10) nor functioning (SOFAS) were associated with substance use for either the UHR or FEP group (see Table 2).

**Table 2:** Odds ratio (OR) of clinical and functional measures associated with the use of different types of substance estimated from multivariate logistic regression

Use (vs. non-use) of		UHR		FEP	
	Predictor	OR (95% CI)	*p*-value	OR (95% CI)	*p*-value
Any illicit substance	BPRS-psychosis	1.05 (0.99–1.11)	0.1	**1.06 (1.02–1.11)**	**0.008**
	BPRS- negative	**0.90 (0.82–0.98)**	**0.022**	**0.92 (0.86–0.99)**	**0.028**
	K10	1.01 (0.99–1.04)	0.2	1.02 (1.00–1.04)	0.1
	SOFAS	0.99 (0.97–1.01)	0.3	1.01 (0.99–1.02)	0.2
ATS	BPRS-psychosis	1.06 (0.98–1.14)	0.2	**1.07 (1.02–1.12)**	**0.004**
	BPRS- negative	**0.87 (0.76–0.99)**	**0.036**	**0.92 (0.86–1.00)**	**0.039**
	K10	1.01 (0.98–1.04)	0.6	1.01 (0.99–1.03)	0.4
	SOFAS	0.99 (0.97–1.01)	0.5	0.99 (0.98–1.01)	0.3
Cannabis	BPRS-psychosis	1.05 (1.00–1.12)	0.07	**1.05 (1.01–1.10)**	**0.025**
	BPRS- negative	**0.90 (0.83–0.99)**	**0.025**	0.94 (0.88–1.01)	0.1
	K10	1.01 (0.99–1.03)	0.2	1.01 (0.99–1.03)	0.2
	SOFAS	0.99 (0.98–1.01)	0.4	1.01 (0.99–1.02)	0.3
Tobacco	BPRS-psychosis	1.05 (0.99–1.11)	0.1	**1.05 (1.00–1.09)**	**0.05**
	BPRS- negative	0.92 (0.84–1.01)	0.07	**0.92 (0.87–0.98)**	**0.01**
	K10	1.01 (0.99–1.04)	0.3	1.00 (0.98–1.02)	0.8
	SOFAS	1.00 (0.98–1.01)	0.7	0.99 (0.98–1.01)	0.3

*P* values in bold signify a statistically significant difference

Odds ratio (OR) of substance use (dependent variable) associated with one unit increase in clinical and functional measures (independent variable) estimated from multivariate logistic regression model controlling for confounding factors including age, gender, sexual orientation, Aboriginal and Torres Strait Islander (ATSI), and culturally and linguistically diverse (CALD) status. Missing data were imputed via MICE using 20 imputed datasets

^a^illicit substance includes cannabis, amphetamine-type stimulants (ATS), cocaine, hallucinogens, and opioids (i.e. does not include the non-medicinal use of pharmaceuticals)

### Network analysis of substance use in each cohort

**Fig. 1:**
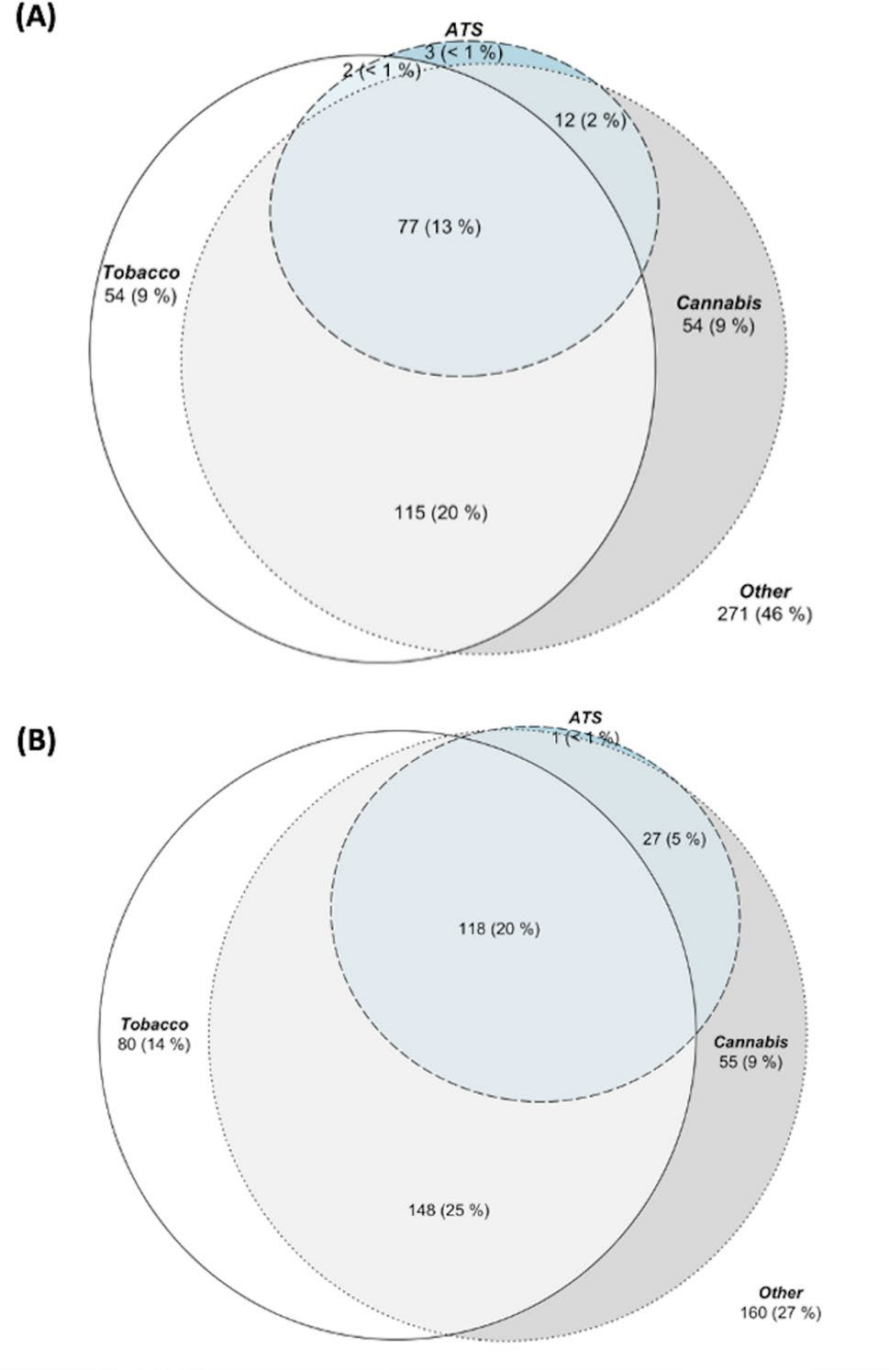
Venn diagram of prevalence of using tobacco, cannabis, and amphetamine-type stimulants (ATS) for the **A** UHR cohort and **B** FEP cohort

**Fig. 2:**
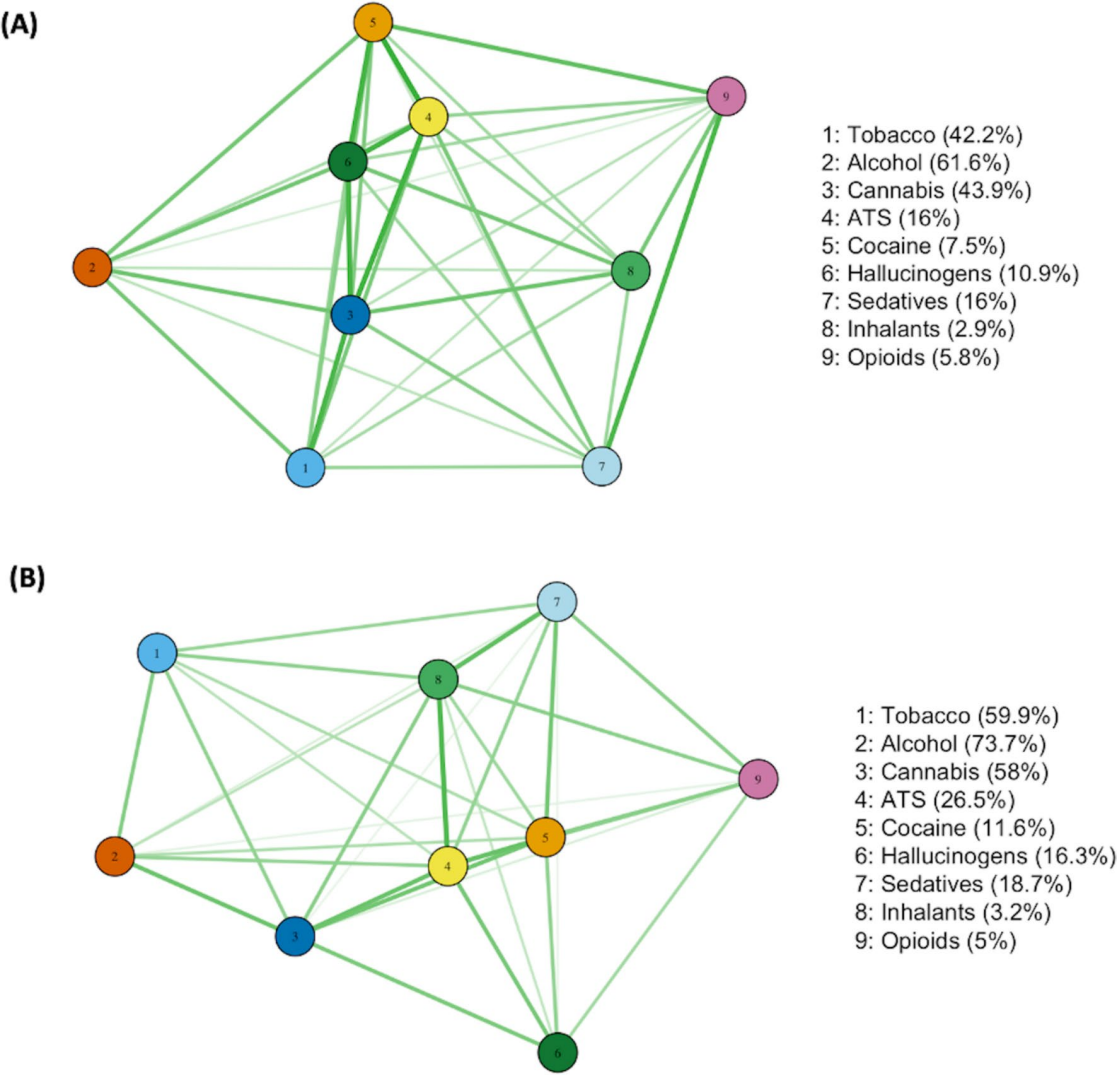
Network analysis of use of substances for the **A** UHR cohort and **B** FEP cohort. Note: Proximity between substance nodes indicates relative strength of positive correlation in usage

The high co-use of multiple substances, particularly tobacco and cannabis, is shown in Fig. 1. Network analysis of the interrelations between substances used in the two different treatment groups (Fig. 2) revealed the qualitative differences in patterns of substance use between the UHR and FEP cohorts. Overall substance use in the UHR group showed stronger associations between the use of each drug and the use of another. On the other hand, drug use in the FEP cohort showed slightly weaker associations between each other as a result of diverse choices in polysubstance use in this group (the median number of types of drug used in the FEP group is 3 compared with 2 in the UHR group, see Table S2).

## Discussion

### Summary of findings

A high prevalence of the past 3-month substance use was observed in both cohorts, with the use of ATS, cannabis, and tobacco all associated with significantly higher levels of positive symptoms in the FEP group, a finding consistent with extant literature [6, 12, 29]. Conversely, in the UHR group, substance use was associated with reduced negative symptoms. There were similar effect sizes and directions in positive and negative symptoms across the different substances, including tobacco. This convergence of associations is expected in the setting of high co-occurrence of use; for example, 20% of the UHR cohort used tobacco and cannabis, but only 9% used cannabis alone. Concomitant with the pronounced overlap in the use of ATS, tobacco, and cannabis is a homogeneity of presentation among those who use them, with each substance tending towards a clinical picture of more florid positive psychotic symptoms with diminished negative symptoms. Effect size was generally diminished in the UHR cohort, where no associations with positive symptoms were observed. No association was found between substance use and functioning nor psychological distress in either cohort.

Network analysis demonstrates particularly strong intercorrelations between the use of ATS and the use of all other studied substances, suggesting that ATS use may be useful as a marker of risky polysubstance use in early psychosis populations, a finding that merits further investigation but is supported by research in the general population [17]. Weaker associations between the use of substances in the FEP group suggest that substance usage is more spread across a variety of substances. On the other hand, people who used ATS, cocaine, and hallucinogens in the UHR cohort were more tightly grouped. This suggests that intervention in substance use in those who are at UHR status could mitigate a trend towards polysubstance use and polysubstance use disorder in those who go on to experience a FEP, with higher rates of polysubstance use in this group represented in Fig. 1.

### Substance use prevalence between cohorts and demographic data

A significantly larger proportion of the FEP population was found to have used any illicit substance, tobacco, cannabis, and ATS compared to the UHR cohort. Substance use prevalence in the population sampled here falls within prevalence rates seen in extant literature with 44% of the UHR cohort and 58% of the FEP cohort found to use cannabis; in papers analysed in a review on the topic [2], cannabis use prevalence was found to vary between 33 and 54% for UHR groups and 13 and 64% for FEP populations. The next most common substances used were ATS, a finding echoed by a similar Australian FEP cohort [10].

### Substance specific effects, symptoms and functioning

The use of any illicit substance and of ATS were associated with significantly lower negative symptoms in both cohorts and additionally higher levels of positive symptoms for the FEP cohort only. An association between higher levels of positive symptoms of psychosis and substance use is well established [26, 27]. Relationships between negative symptoms of psychosis and substance use are less consistent in the literature, varying with study methodology employed and cohorts analysed.

Frequency and persistence of substance use are both important clinical variables; however, frequency of substance use for an individual can vary substantially alongside changes in life circumstances. Dichotomising substance use to a single Yes/No within the 3 months prior to presentation casts a broader net in identifying use among this large cohort. The significant differences in symptomatology observed with this method are less likely to be due to acute intoxication or withdrawal which may be seen in people who use regularly at the time of assessment. Therefore, it may be more likely to reflect underlying differences in cohorts between those who do and do not use these substances.

No significant correlation between substance use and functioning or levels of psychological distress were seen. Past studies of FEP populations have also not demonstrated any consistent relationship between measures of functioning and substance use [28]. However, persistent substance use has been specifically studied in an FEP population by excluding individuals who sporadically use substances or who cease using after baseline assessment and before follow-up [36]. Using this method, persistent use was found to be associated with poorer functioning. Given that the method used in this current analysis did not discriminate between persistent and sporadic use the absence of any correlation between substance use and functioning is potentially unsurprising.

Tobacco use predicted higher levels of positive symptoms and lower of negative symptoms in the FEP cohort. This association arises in the setting of a high co-occurence of tobacco use and that of other substances, particularly cannabis. A meta-analysis probing symptomatological correlations of cannabis and nicotine in adults with known psychotic illness found elevated positive symptoms in nicotine *and* cannabis users, but no elevation in positive symptoms for those who used nicotine only [29], suggesting that this association may be due to the cannabis used by tobacco smokers in the FEP cohort.

### Network analysis

Network analysis used in this paper demonstrates that ATS use is more strongly associated with the use of all other surveyed substances than any other substance category. The relationship between ATS and polysubstance use in FEP populations has been demonstrated previously with polysubstance use found in 50% of those who used cannabis but 65% of those who used cocaine/amphetamines in a Canadian cohort [28].

It further demonstrates a difference in the pattern of substance use between cohorts. While the UHR group tends to have tighter grouping and stronger associations between the use of “hard” drugs, the FEP group shows more laxity of correlation, with more varied patterns of substance use. This indicates a spreading in the pattern of substance use as age and psychotic illness progresses with more varied pattern of substance use. Increased polydrug use in FEP compared to UHR groups is quantified in Fig. 1 which shows, for example, identical proportions of people in UHR and FEP using cannabis or ATS alone (9% & < 1%, respectively), but a doubling in the proportion using both cannabis and ATS in the FEP group (2% UHR, 5% FEP).

### Strengths and Limitations

A strength of these analyses is that with a consent rate of 92%, the data are highly representative of young people presenting to these real-world early intervention in psychosis services. Data comprises two well matched cohorts from appreciably similar demographic and geographical backgrounds, permitting effective comparison between the UHR and FEP groups.

Limitations, however, also must be considered. Firstly, young people using a variety of substances were not separated from those using each substance individually. The similarities seen in the symptomatology of groups using a given substance were therefore confounded by the use of other surveyed substances. Research intending to comment on substance-specific effects, for example between tobacco and psychotic illness, could compare nonusers with tobacco-only users and those who use tobacco and other substances. Polydrug use is so high, however, that dissecting out individual substances becomes difficult due to the statistical requirement of large samples. Secondly, results seen here may not be generalisable outside of Australia given that substance use patterns differ by geography. Thirdly, data analysed relied on routine clinical data collection completed by clinicians and may therefore not be as accurate as that collected in research studies, as there were no specific reliability checks. Client under-reporting of substance use may be common. Although the quality of the MDS data is monitored, the data still suffer from quality issues including data that is missing or subject to entry errors. Finally, the cross-sessional nature of the data does not allow us to evaluate how substance use trajectories may impact on development and progression of clinical symptoms and how different risk factors (e.g. demographics) may mediate or modify the association.

## Implications

Findings demonstrate similar symptom profiles of higher levels of positive and lower levels of negative symptoms among those who use all surveyed substances, including tobacco. Current understandings of tobacco use in psychotic illness suggest it is a marker of illness severity, which may also contribute directly to pathogenesis [20, 23]. That tobacco use in early psychotic illness was found to correlate similarly with symptomatology as other substances known to contribute to pathogenesis begs the question of whether early cessation of tobacco use could also associated with lower negative symptoms at 10 years [38]. Clinician attitude has been found to be the primary predictor of treatment for tobacco use being available to patients in community mental health settings [30]. Simple interventions aimed toward changing clinicians’ attitudes therefore have the potential to reduce tobacco use among early psychosis and at-risk populations. In addition, limited research has investigated effective smoking cessation interventions for people with early psychotic illness. It is unknown whether standard treatment paradigms are effective in this population, and future research could investigate specific methodologies for people with early and emerging psychotic illness.

The associations shown between ATS and the use of all other substances suggested that use of this substance may be useful as a simple binary marker of potentially risky substance use. Further research could focus specifically on the substance use patterns of those who use ATS and those who do not to further ascertain the utility of this marker. Given that clinicians are routinely asking about the use of each substance through this MDS, recognising and subsequently acting on ATS use throughout a young person’s time in service could provide the opportunity to change what may be a trajectory towards poor outcomes.

The results of this study moreover suggested that both in the domain of tobacco use and substance use more broadly, the involvement of people at UHR of developing psychosis with early intervention services represents a critical opportunity for intervention. This was suggested both by network analysis showing a more limited and focussed pattern of substance use in this group, and by the absence of exacerbated positive symptom associations. Specific interventions tailored to this group could 1) prevent polysubstance use type patterns emerging that are seen in FEP; 2) mitigate the worsened positive psychotic symptoms seen in substance using people with FEP, and 3) mitigate the disabling and difficult to treat negative symptoms arising with the progression of psychotic illness that is seen with persistent comorbid substance use [38]. Newly inclusive and expansive treatment efforts incorporating at-risk youth alongside further innovation in alcohol and drug treatment integration are just cause for hope among staff and patients in this dynamic clinical space. Presentation to early psychosis services appears to represent a key opportunity for early intervention and treatment for substance use problems in UHR and FEP. Further research is needed to guide the identification and targeted treatment of those likely to continue substance use despite their engagement in early psychosis programmes.

## Supplementary Information

Below is the link to the electronic supplementary material.

Supplementary file1 (DOCX 357 KB)

## Data Availability

The data underlying these analyses are owned by a third party, headspace. The authors do not have the authority to share or distribute the third-party data. headspace requires users to provide formal agreement and ethical clearance to regulate data storage and use. Other researchers wishing to access the data can contact the corresponding author to liaise with data access approvals.
