# Supplementary material for: Correlates of substance use in a large naturalistic cohort of young people with early and emerging psychosis
Source: Soc Psychiatry Psychiatr Epidemiol. 2023 Feb 20;58(10):1447–56. doi: 10.1007/s00127-023-02436-w (PMC10460356; doi:10.1007/s00127-023-02436-w)
Supplement: Supplementary file 1 — Supplementary file1 (DOCX 357 KB) [file 127_2023_2436_MOESM1_ESM.docx]

Supplementary Information

**Table S1** Patient characteristics and clinical outcomes of UHR and FEP cohorts

| Characteristic |  | UHR  N= 609 | FEP  N=643 |
| --- | --- | --- | --- |
| Age at intake | Median (Q1-Q3) | 17 (15, 20) | 20 (18, 22) |
| Gender |  |  |  |
| Female | n (%) | 317 (52%) | 201 (31%) |
| Male | n (%) | 273 (45%) | 434 (68%) |
| Non-binary | n (%) | 14 (2.3%) | 5 (0.8%) |
| Sexual orientation | n (%) |  |  |
| Heterosexual/straight | n (%) | 397 (65%) | 443 (73%) |
| Other | n (%) | 188 (31%) | 167 (27%) |
| First Nations | n (%) | 54 (8.9%) | 52 (8.3%) |
| CALD | n (%) | 82 (13%) | 161 (26%) |
| BPRS-Psychosis | Median (Q1-Q3) | 8 (6, 10) | 9 (6, 1) |
| BPRS-Negative | Median (Q1-Q3) | 5 (3, 7) | 5 (3, 8) |
| K10 | Median (Q1-Q3) | 32 (25, 38) | 24 (17, 30) |
| SOFAS | Median (Q1-Q3) | 56 (50, 65) | 55 (50, 65) |

Missing data for UHR include 1 for age at intake, 5 for gender, 24 for sexual orientation, 10 for ATSI, 11 for CALD, 143 for BPRS-psychosis, 143 for BPRS-negative, 71 for K10 and 20 for SOFAS. Missing data for FEP include 3 for gender, 33 for sexual orientation, 15 for ATSI, 25 for CALD, 172 for BPRS-psychosis, 172 for BPRS-negative, 113 for K10 and 49 for SOFAS. Acronyms: CALD- culturally and linguistically diverse. BPRS- Brief psychosis rating scale. SOFAS- social and occupational functioning assessment scale. K10- Kessler 10 scale of psychological distress. UHR- ultra-high risk. FEP- first episode psychosis

**Table S2** Drug use patterns in UHR and FEP cohorts

| Characteristic |  | UHR  N= 609 | FEP  N=643 |
| --- | --- | --- | --- |
| Drug use patterns |  |  |  |
| No drug use | n (%) | 173 (29%) | 100 (17%) |
| Use one type of drug | n (%) | 110 (19%) | 71 (12%) |
| Use multiple types of drugs | n (%) | 304 (52%) | 429 (72%) |
| Types of drugs used | Median (Q1-Q3) | 2 (0-3) | 3 (1-4) |

Missing data: 22 in the UHR group and 43 in the FEP group.

| (A) | 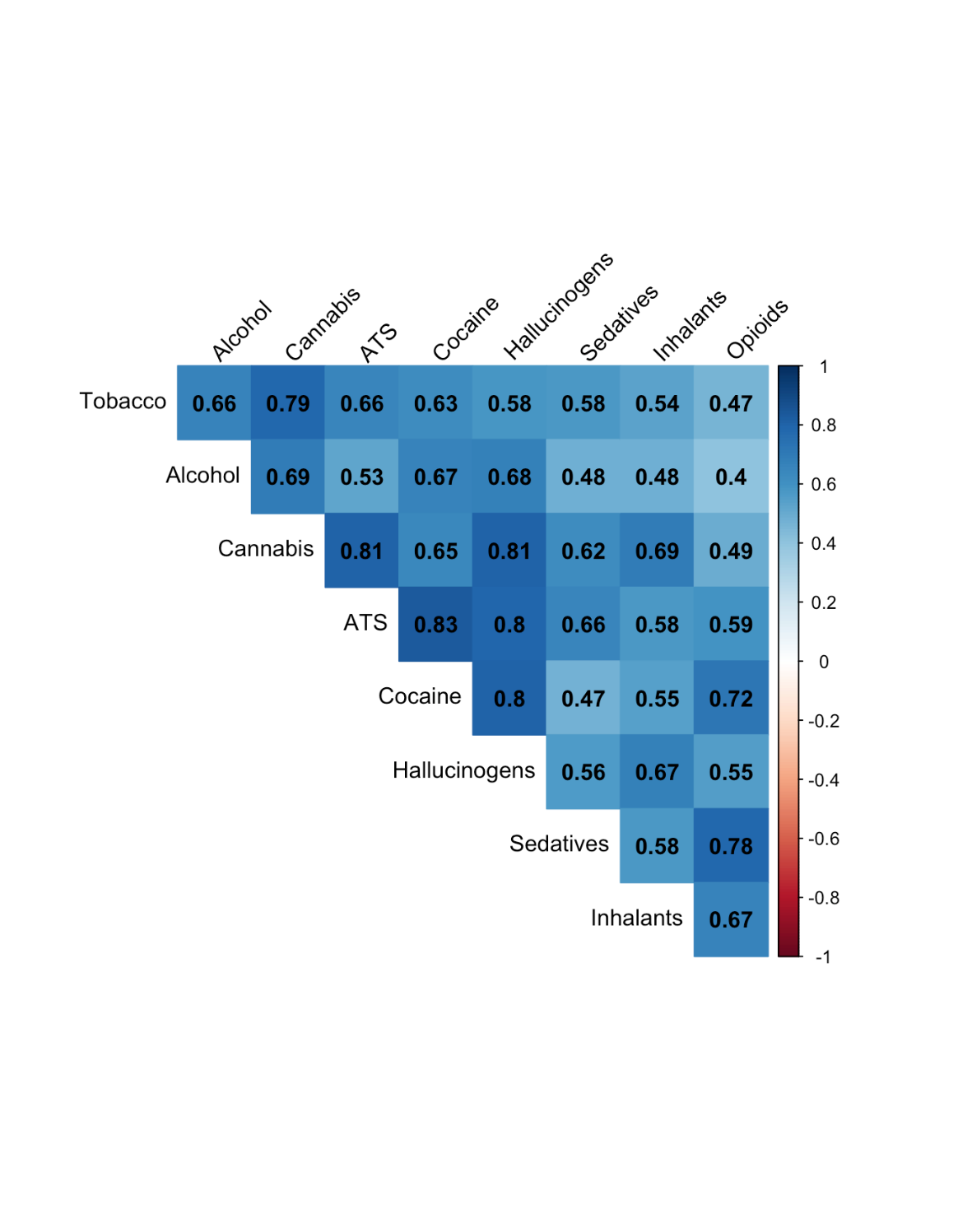 |
| --- | --- |
| (B) | 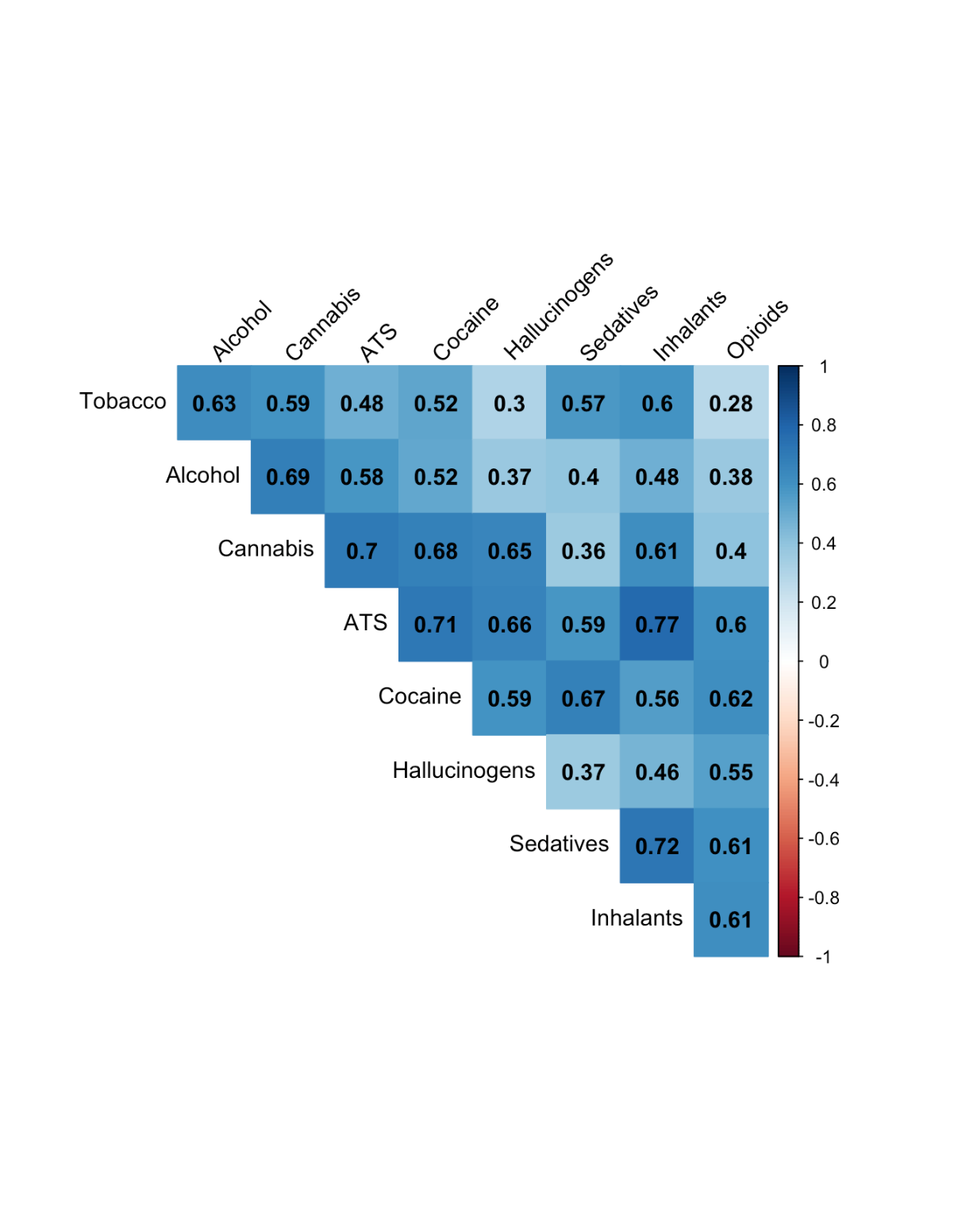 |

Figure S1 Tetrachoric correlations ($r_{t}$) between use of different substance for (A) UHR cohort and (B) FEP cohort
